# On-Flow Synthesis of Co-Polymerizable Oligo-Microspheres and Application in ssDNA Amplification

**DOI:** 10.1371/journal.pone.0159777

**Published:** 2016-07-22

**Authors:** Se Hee Lee, Jae Ha Lee, Ho Won Lee, Yang-Hoon Kim, Ok Chan Jeong, Ji-Young Ahn

**Affiliations:** 1 Department of Microbiology, Chungbuk National University, 1 Chungdae-Ro, Seowon-Gu, Cheongju 28644, South Korea; 2 Graduate School of Mechanical Engineering, Inje University, 197 Inje-ro, Gimhae, Gyungnam 621–749, South Korea; 3 Department of Biomedical Engineering, Inje University, 197 Inje-ro, Gimhae, Gyungnam 621–749, South Korea; University of Helsinki, FINLAND

## Abstract

We fabricated droplet-based microfluidic platform for copolymerizable microspheres with acrydite modified DNA probe. The copolymerizable 3-D polyacrylamide microspheres were successfully produced from microcontinuous-flow synthesis with on-channel solidification. DNA copolymerization activity, surface presentation and thermostability were assessed by using fluorescent labeled complementary probe. The binding performance was only visible on the surface area of oligo-microspheres. We show that the resulting oligo-microspheres can be directly integrated into a streamlined microsphere-PCR protocol for amplifying ssDNA. Our microspheres could be utilized as a potential material for ssDNA analysis such as DNA microarray and automatic DNA SELEX process.

## Introduction

Functionally active nucleic acids, including aptamers [1] and single-strand oligonucleotide ligands (single stranded DNA, ssDNA), have been extensively used as target recognition elements due to their intrinsic properties. To especially carry out the ssDNA analysis, PCR amplification should be followed by the conversion of dsDNA to ssDNA using the extra steps such as asymmetric PCR [2], biotin-streptavidin separation using magnetic beads [3], enzymatic degradation, and denaturation of dsDNA by heating or by alkaline treatment, etc. [4–7]. However, each of these methods has limitations in terms of the requirement of highly trained experts, expensive enzyme usage, and inadequate productivity. Therefore, the development of high-throughput, simple and cost effective methods that can overcome the limitations are essential.

Recent advances in microfluidic techniques have enabled the use of microspheres in molecular biological experiments [8]. Microspheres are three-dimensional (3-D) polymer networks with a cross-linked structure. Such structure can be used a new high-throughput platform for diagnostics, drug screening, encapsulation of bioelements such as DNA, enzyme, and cells including bacteria, and biosensor elements [9,10]. Currently, applications of microfluidic device involve the manipulation of water-in-oil microspheres to provide lower cost alternative experimental format [11,12].

Several innovative processes for the synthesis of micrometer-scale polymer particles have been successfully demonstrated using microfluidic devices. Droplet-based microfluidics enables both reliable production of highly homogeneous microspheres and control of the manipulation process, thus providing controlled size and morphology of obtained microspheres [13,14]. This attractive feature has been regarded as a fundamental strategy for achieving the high throughput manner and developing customized microspheres tailored to users. Meanwhile, they are competitive or even far superior to the conventional Inverse-Suspension (IS) technology [8]. However, this bulk technique is realistically laborious, time-consuming with three to four steps, and highly susceptible to manufacture environments including stirring rate, surfactants, and temperature.

**Fig 1:**
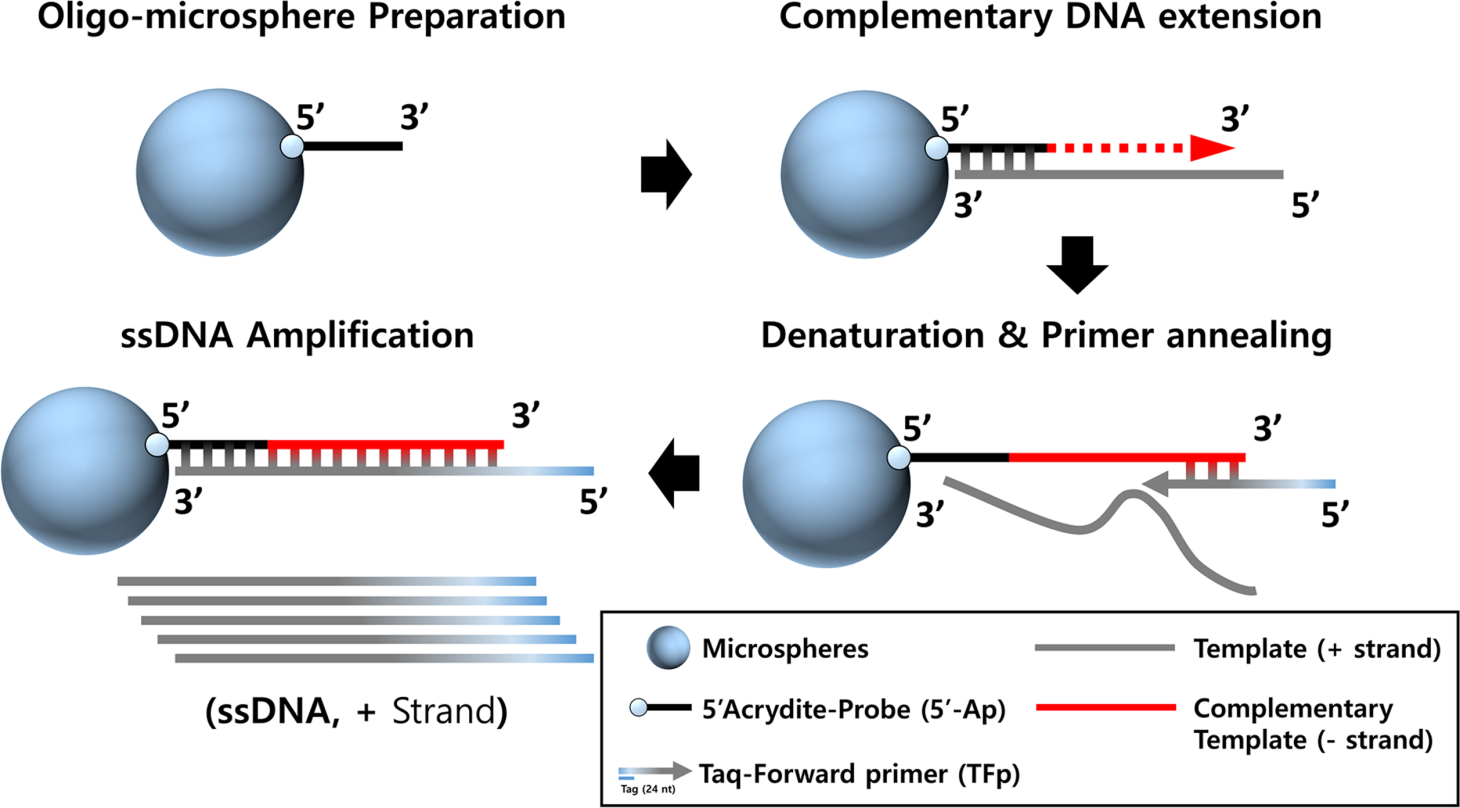
A graphical summary of the Microsphere-PCR protocol. The Microsphere-PCR synthesizes ssDNA copy of a DNA template.

In this study, we fabricated a droplet-based microfluidic platform to produce polyacrylamide microspheres with acrydite modified oligonucleotides DNA probes. The main challenges of using polyacrylamide support are high thermal stability, high attachment capacity, and low non-specific absorption levels [15]. Polyacrylamide microspheres carrying ssDNA probes at their periphery can be readily synthesized using the droplet-based microfluidic platform as a solid support. The 3-D configuration of the microspheres and DNA immobilization were evaluated to determine the complementary interaction between ssDNA probes and their fluorescent labeled complement partner. These oligo-microspheres allowed us to achieve ssDNA amplification. Another motivation to use polyacrylamide-based supports was the co-polymerizable property of acrydite modified DNA probe. Since each acrydite modified DNA probe is polymerizable with acrylamide monomer and bis-acrylamide, DNA probe can be covalently incorporated into polyacrylamide microsphere compositions and attached to the support at multiple sites during polymerization [16]. DNA extension and covalent immobilization of the functionalized microspheres was analyzed for their ability in DNA complement binding to the microspheres. **Fig 1** shows the graphic concept and process of ssDNA amplification. Short DNA probes, chemically modified with a polymerizable acrylamide group (5’Acrydite-Probe, Ap), were homogeneously mixed with acrylamide monomer and introduced into the microfluidic device. In the presence of oligo-microspheres, the DNA template (plus (+) strand) can assemble with DNA probes on the surface of microspheres. Complementary DNA extension (minus (-) strand) and subsequent ssDNA amplification (plus (+) strand) can be performed in a single-tube reaction using forward primer only, resulting in streamlined microsphere-PCR protocol. Therefore, our strategy makes it possible to acquire ssDNA amplicons without dsDNA separation or removal. It is expected that the use of oligo-microspheres can be extended to be applied in an automatic SELEX and other ssDNA analytic assays such as DNA-microarray, mutation typing and SNP experiments.

## Materials and Methods

### Oligonucleotides

Oligonucleotides containing 5’-terminal Acrydite groups (Acrydite probe, Ap) and 5’-Cy3-labeled complementary oligonucleotides (complementary Acrydite probe, cAp) were obtained from Integrated DNA Technologies, Inc. (IDT, Coralville, IA, U.S.). Unmodified template DNA (T), template DNAs (Random Library, 76 nt DNA template, T7 Linked 76 nt DNA template, Complementary 107 nt DNA template) and primers were obtained from Bioneer (Daejon, Korea). Lyophilized oligonucleotides were dissolved in TE buffer (10 mM Tris-HCl, pH 8.3, 1 mM EDTA) and stored frozen at -20°C. Concentrations were determined based on A260 nm readings (assuming 33mg/mL oligonucleotide per 1 optical density [OD] unit). All concentrations were referred to oligonucleotide strands. Sequences of oligonucleotides used in this study are listed in **Table 1**.

**Table 1:** Sequences of oligonucleotides used to amplify ssDNA based on microsphere-PCR.

Name	Sequence (5’ to 3’)	Size (nt)
Template (N40 Random library)	ATACCAGCTTATTCAATT(N_40_)AGATAGTAAGTGCAATCT	76
76 nt DNA template	ATACCAGCTTATTCAATTCCAAAAGCGCACCCATATATGTTCTATGTCCCCCACCTCGAGATAGTAAGTGCAATCT	76
T7 Linked 76 nt DNA template	TTTTTTTAGATTGCACTTACTATCTCGAGGTGGGGGACATAGAACATATATGGGTGCGCTTTTGGAATTGAATAAGCTGGTAT	83
Complementary 107 nt DNAtemplate	GGTAATACGACTCACTATAGGGAGATACCAGCTTATTCAATTCCAAAAGCGCACCCATATATGTTCTATGTCCCCCACCTCGAGATAGTAAGTGCAATCTAAAAAAA	107
5’-Acrydite Probe (5’-Ap), T7 Linker*	Acrydite-TTTTTTTAGATTGCACTTACTATCT	25
T primer	TTTTTTTAGATT	12
Complementary probe (cAp)-Cy3	Cy3-AGATTGCACTTACTATCT	18
^**†**^ Tag-Forward Primer (TFp)	GGTAATACGACTCACTATAGGGAGATACCAGCTTATTCAATT	42
Reverse primer (R)	AGATTGCACTTACTATCT	18

* T7 Linker: TTTTTTT

^†^ Tag: GGTAATACGACTCACTATAGGGAG

### Fabrication of PDMS microfluidic platform

**Fig 2:**
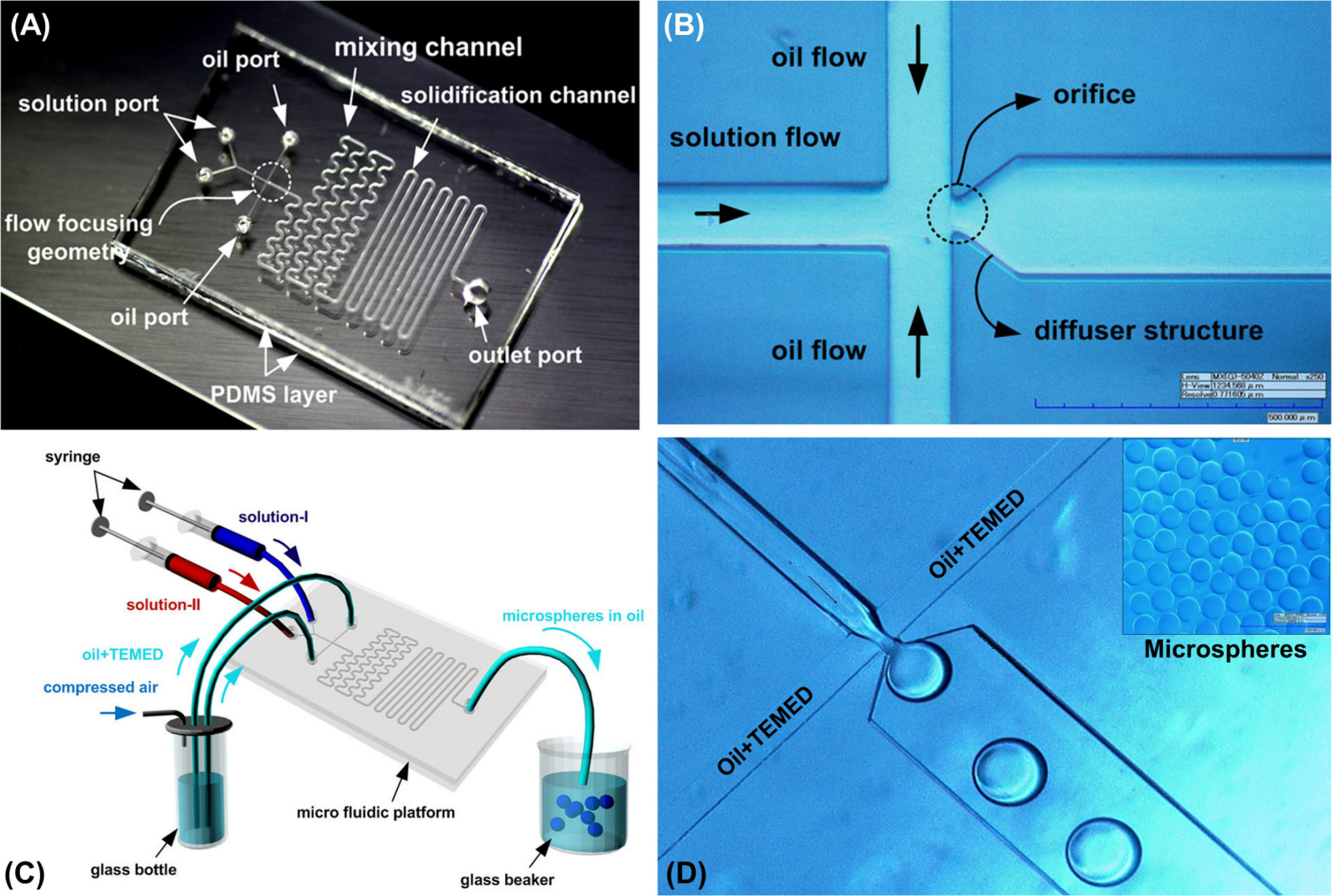
Fabricated polymeric droplet-based microfluidic platform and on-flow microsphere synthesis. (A) Droplet-based microfluidic platform, (B) Flow-focusing geometry. (C) Overall experimental set-up for producing polyacrylamide microspheres. (D) On-flow synthesis of polyacrylamide microsphere in the main channel. The inset figure shows the ccaptured image being formed microspheres.

The microfluidic platform for the production of microsphere was fabricated using standard replica molding process with polydimethylsiloxane (PDMS). First, liquid PDMS (Sylgard 184, Dow Corning Inc.) mixed with the base polymer and curing agent in the ratio of 10:1 was poured onto a prepared SU-8 mold for microfluidic network. After centrifugation at 1500 rpm for 5 seconds, they were cured at 75°C for 30 minutes. Liquid PDMS for the flat PDMS layer was poured onto glass substrate and cured. The cured PDMS layer for microfluidic channel network was peeled off from the SU-8 mold manually, and then bonded with the prepared flat PDMS layer using the atmosphere plasma bonding method [17]. The structure of the microchannel is shown in **Fig 2**.

### Production of oligo-microspheres

The polyacrylamide microspheres containing oligonucleotide Acrydite probes (Ap, 5’-Acrydite-(linker sequence, 7 mer) AGA TTG CAC TTA CTA TCT-3’) were based on the copolymerization of acrylamide monomer-modified oligonucleotides [15]. Solution I contained 10% acrylamide:bis solution (19:1) and 100 μM oligonucleotide; Solution II contained 40% ammonium persulfate. Two microsyringe pumps were used to introduce the continuous phages (Solution I and Solution II) into the microfluidic device, with a flow-rate of 1 mL/h. The microspheres droplets were generated along with the mineral oil containing 0.4% TEMED (*N*,*N*,*N*′,*N*′-Tetramethylethylenediamine, Sigma-Aldrich). The mixed solution droplets gelled into polyacrylamide microspheres in a wavy channel by the polymerization reaction. The flow-rate of the continuous oil flow was 13.35 mL/hr while the 60 kPa of the compressed air pressure was applied into the glass bottle using the lab-designed pneumatic control system [18]. Oligo-microspheres were collected from the glass bottle with a large bore pipet tip and washed very carefully in deionized water. The microspheres were finally resuspended in TE buffer (pH 8.0) to an average concentration of 25 microspheres/100 μl. They were stored at 4°C for up to 2 weeks. Observation of microspheres was carried out with a microscope at 100 X magnification.

### DNA hybridization on the surface of Oligo-microsphere

Complementary oligonucleotides (DNA probes) attached with fluorescent molecule at 5’-end (Cy-3 labeled, cAp) were used to verify the oligonucleotide attachment on the surface of the microsphere. Hybridization reaction was performed using 1.5 mL microtube. For complementary hybridization reaction, ~ 10 microspheres was randomly filled in 100 μL of TE buffer and incubated at 42°C for 5 min. After adding 1 pmol/ μL of cAp into the reaction tube, the mixture was incubated at 42°C for 1 hr. the final hybridization volume was 50 μL. After the 1 hr incubation, the microspheres were washed three times in 500 μL of TE solution. Results of hybridization were imaged under a confocal microscope (LSM 510, Carl Zeiss). To verify the thermal attachment stability, PCR amplifications with various thermal cycle conditions (5, 10, 15, 20, 25 and 30 rounds of amplification performed at 95°C for 30 s, 52°C for 30 s, and 72°C for 30 s) was performed. These microspheres were then washed with distilled water several times and incubated with cAp in an identical volume of reaction buffer at room temperature for 1 hr. Thereafter, the fluorescence activities were measured by using 96-well black plate (Costar®, Sigma-Aldrich) on a FLx800™ Multi-Detection Microplate Reader (Bio-Tek).

### Generation of ssDNA amplicons by microsphere-PCR

Microsphere-PCR reactions were performed in 50 μL of reaction volume. Acrydite-probe (Ap) sequence used was 5’-Acrydite-(linker sequence, 7 mer) AGA TTG CAC TTA CTA TCT-3’. The template sequence was as follows: 5’- ATA CCA GCT TAT TCA ATT (Random sequence, 40 mer) AGA TAG TAA GTG CAA TCT-3’. Tag (24nt, underline)-forward primer (TFp) used was 5’- GGT AAT ACG ACT CAC TAT AGG GAG ATA CCA GCT TAT TCA ATT-3’. PCR mixture contained ~ 10 Ap-microspheres, 0.02 μM of template, 0.2 μM of Tag-Forward primer (TFp), 5 μl of Taq buffer, 2.5 μM of dNTP each, and 1 Unit of Ex taq (Takara, Japanese). PCR amplification was performed for 25 cycles under following condition of temperature: 95°C for 4 min, 95°C for 30 s, 52°C for 30 s, and 72°C for 30 s. After PCR amplification, 6× loading buffer was added and 15 μl of each sample was run on at 100 V for 35 min on a 2% agarose gel in 0.5X TAE buffer. Gel Doc™ EZ System was used to analyze the agarose gel result.

### Asymmetric PCR condition

PCR conditions for asymmetric PCR were exactly the same as those of symmetric PCR except that the ratios of primers used were different. Asymmetric PCR was carried out in a volume of 50 μL with Tag-forward primer (TFp, 5’-GGT AAT ACG ACT CAC TAT AGG GAG ATA CCA GCT TAT TCA ATT-3’) and reverse primer (R, 5’-AGA TTG CAC TTA CTA TCT-3’). PCR mixture contained 0.02 μM of identical template, 0.4 μM of Tag-F primer, 0.02 μM of R primer, 5 μL of Taq buffer, 2.5 μM of each dNTP, and 1 Unit of Ex taq (Takara, japenese). PCR amplification was performed 25 cycles under following conditions: 95°C for 4 min, 95°C for 30 s, 52°C for 30 s, and 72°C for 30 s.

### Real-time PCR and melting Tm analysis for dsDNA hybrids

The microsphere-PCR was performed in the presence and absence of a 76 nt DNA template (0.02 μM, 5’-ATA CCA GCT TAT TCA ATT CCA AAA GCG CAC CCA TAT ATG TTC TAT GTC CCC CAC CTC GAG ATA GTA AGT GCA ATC T -3’). Following microsphere-PCR, the resultant microspheres were extensively washed with nuclease-free DW 5 times. To prove the formation of dsDNA on the surface of microspheres, real-time PCR amplification and melting Tm analysis were carried out. Amplification was performed in a final reaction volume of 20 μl, containing 1x iQ ^TM^ SYBR® Green supermix (Bio-rad, California, USA), 0.02 μM of T primers, 0.02 uM of Tag-Forward primers (TFp) and the dsDNA hybrids to be amplified (Reference Duplexes (RD)—83 nt/107 nt DNA hybrids, and microsphere-PCR dsDNA byproducts—83 nt/107 nt hybrids). The amplification was carried out using the Rotor-Gene Q (QIAGEN Hiden, Germany) and the cycle conditions were as follows: 95°C 10 min, followed by 40 cycles of 95°C 10 s, 52°C 15 s, 72°C 20 s. With regard to the synthesized reference duplexes (RD), complementary single-strands (83 nt/107 nt hybrids) were combined in 1:1 molar ratio (10 pmole each), heated to 95°C for 5 min and slowly cooled down to room temperature for ∼5 min to form DNA hybrids that can be thermal cycled using real-time PCR protocol. After amplifying, a melting Tm analysis was automatically performed from 55°C to 95°C at 1°C /s melt rates. The melting temperature (Tm) was defined as the highest peak of the curve.

## Results and Discussion

### Microfluidic platform for generating microspheres

Droplet based microfluidic platform was used to fabricate polyacrylamide microspheres. **Fig 2A** shows that the fabricated polymeric droplet-based microfluidic platform is consisted of two PDMS layers. The upper PDMS layer contained microfluidic channel network for generating microsphere. There are three kinds of fluidic channels in microfluidic network: 1) flow focusing geometry for droplet generation of introduced solution flow as dispersed phase with mineral oil as continuous phase, 2) serpentine channel for mixing procedure of two mixed solutions in the single microsphere, and 3) sequential polymerization channel for solidification. The bottom one was flat PDMS layer without any replicated structure. It was used to form hydrophobic channel surfaces using the natural hydrophobic property of PDMS to prevent solution flow from adhering to the surrounding fluidic channel walls. The height of the microchannel structure was 125 μm. The lengths of the channels for mixing and polymerization were 71.7 and 96.4 mm, respectively.

**Fig 2B** shows a magnified view of the flow-focusing junction region. Once two immiscible flows such as a solution flow and mineral oil flows was introduced into the three channels, they are forced to flow through the narrow orifice structure. The two liquid flows were then intersected by the interaction of two fluids with different flowrates and viscosities. Thus, the solution stream can breakup into microspheres [17,18]. The width of the microchannel for introducing two immiscible fluid flows and one for the microsphere stream in the oil flow was 100 μm and 200 μm, respectively. The width and length of the orifice structure for the microsphere formation are 50 and 25 μm, respectively. The angle of the diffuser structure is 37 deg.

### On-flow synthesis of oligo-microspheres

By employing microfluidic platform, we succeeded in fabricating the oligo-microspheres of the order of 2 × 10^2^ μm with high throughput and high uniformity. The experimental set-up for continuous generation of polyacrylamide microspheres is shown in **Fig 2C and 2D**. Two syringe pumps were used for the generating the to-be-disperse d flow of two solutions (Solution I and II) having flowrates 1 mL/hr. In the case of the oil flow containing TEMED, the lab-designed pneumatic control system [19] were utilized for introducing continuous phase into microfluidic channels since the liquid flow generated by constant compressed air was relatively stable than the case of a syringe pump driven via stepper motor and ball screw with overshooting and fluctuation in liquid flow [20]. Once the compressed pressure was applied into the glass bottle, the stable and continuous pressure-driven oil flow [21] could be generated. The flow-rate of the continuous oil flow measured at the outlet port was 13.35 mL/hr while 60 kPa of the compressed air pressure was applied into the glass bottle. On-channel polymerization of these solution droplets was carried out in a wavy channel with length 96.4 mm. A beaker containing the mineral oil with TEMED was used to collect the produced microspheres. By stirring with low rotational speed (500 rpm), the liquid in a beaker was agitated to prevent the unwanted merging of microspheres. These microspheres were either stored in the same mineral oil solution or rinsed with TE buffer solution for further PCR experiments.

Captured image of the on-channel polymerized microspheres is shown in **Fig 2D (Inset figure)**. The optical observation confirmed that the on-flow synthesis of microspheres was successfully performed using the fabricated microfluidic platform. The averaged diameter of resulting microspheres was 272 ± 7.1 μm, with size variations less than 2.61%. Thus, it was concluded that the microfluidic platform and the operational method proposed in this work could be reproducible and reliable for generating uniform microspheres to provide experimental consistency.

### Co-polymerization attachment of DNA probe to polyacrylamide microsphere

**Fig 3:**
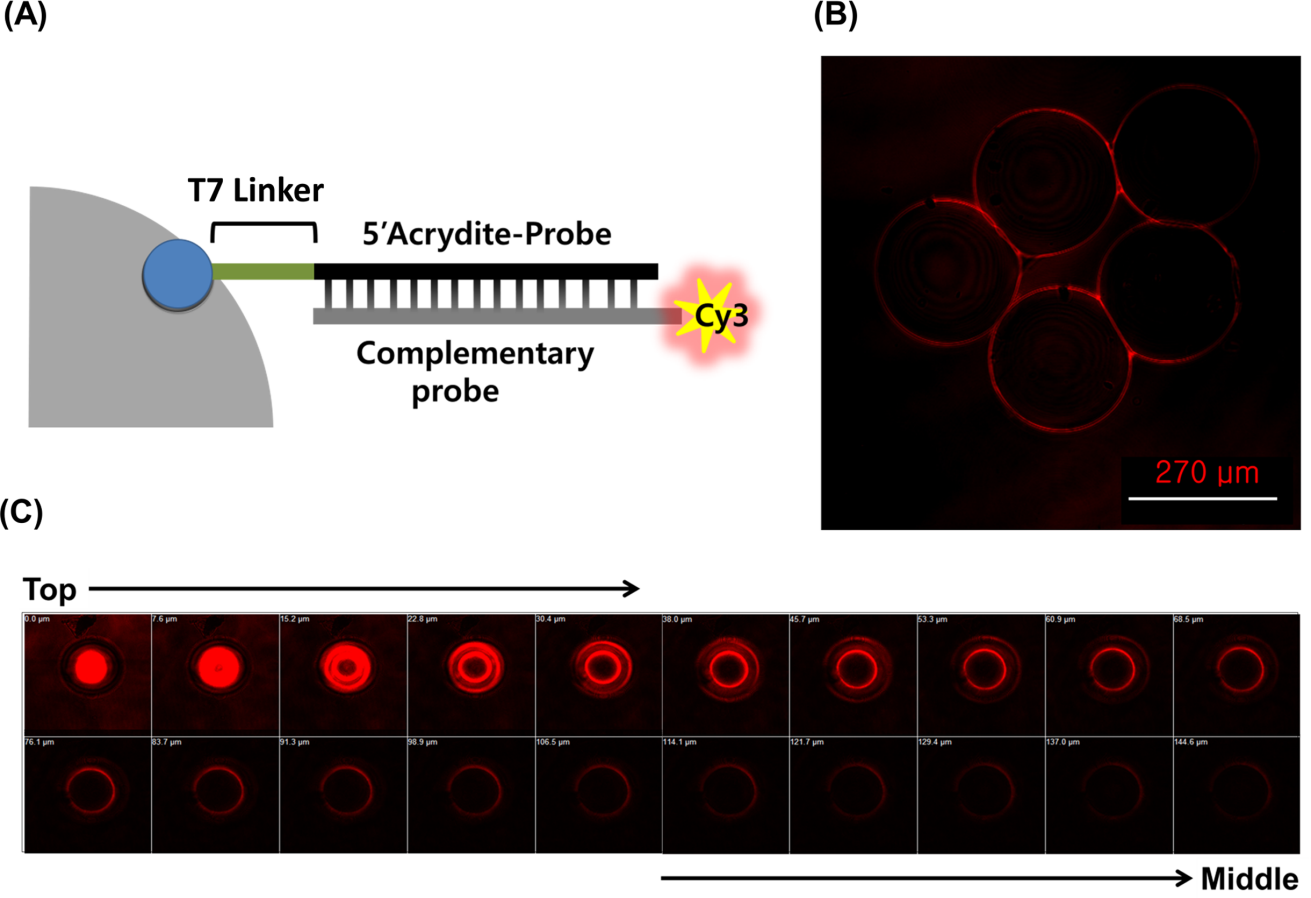
DNA presentation and fluorescence image of probe binding to the surface of 3-D microsphere. (A) The fluorescent signal demonstrated that the oligonucleotides (5’Acrydite-modified DNA probe, Ap) on the surface of the microsphere were capable of hybridizing to the complementary fluorescent labeled DNA probes. (B) The complementary binding performance was visible only on the surface area of Ap-contained microsphere. (C) Z-stack fluorescence images of a single microsphere captured with 3D confocal fluorescence microscopy at different Z-planes. Non-specific interaction or absorption of complementary DNA probes to any of the inside area was not observed in Z-stack fluorescent images. Images were collected at 7.6 **μ**m intervals using a 488 laser.

The functional property of polyacrylamide microspheres was varied by including copolymeriazble payloads along with the polyacrylamide precursor solution. We incorporated the co-polymerized DNA probe into the microsphere solution (solution I) for the purpose of using the microspheres as PCR materials for ssDNA synthesis. To validate the functional attachment of Ap, identical DNA probe with 5’-NH_2_-group instead of 5’-acrytide modification was introduced into the mixture, and tested for Ap contained microspheres in parallel. Following co-polymerization, complementary DNA probe labeled with fluorescent tag, Cy3, was mixed together at room temperature for 1 hr. The reaction samples were subjected to confocal analysis. Due to specific binding between the DNA probe and its corresponding DNA, fluorescence signals were expected to be observed on the microsphere for which their corresponding targets were present. It was confirmed that non-specific incorporation did not take place with 5’-NH_2_-oligo probe (data not shown). The binding performance was only visible on the surface area of Ap-containing microspheres as shown in **Fig 3A** and **3B**. In addition, there was no interference of random-interior orientation, which allowed us to avoid the problem of molecular recruiting into their internal area. **Fig 3C** shows fluorescence images of a single microsphere captured with a confocal laser scanning microscopy. Fluorescence images were collected at 7.6 μm intervals along the **Z**-axis of a microsphere from top plane to 144.6 μm. Results in **Fig 3** indicated that DNA complementary binding occurred in the surface region accessible for PCR and molecular presentation in complex 3-dimensional (3-D) arrangement.

### Microsphere-PCR amplification for single strand DNA (ssDNA)

**Fig 4:**
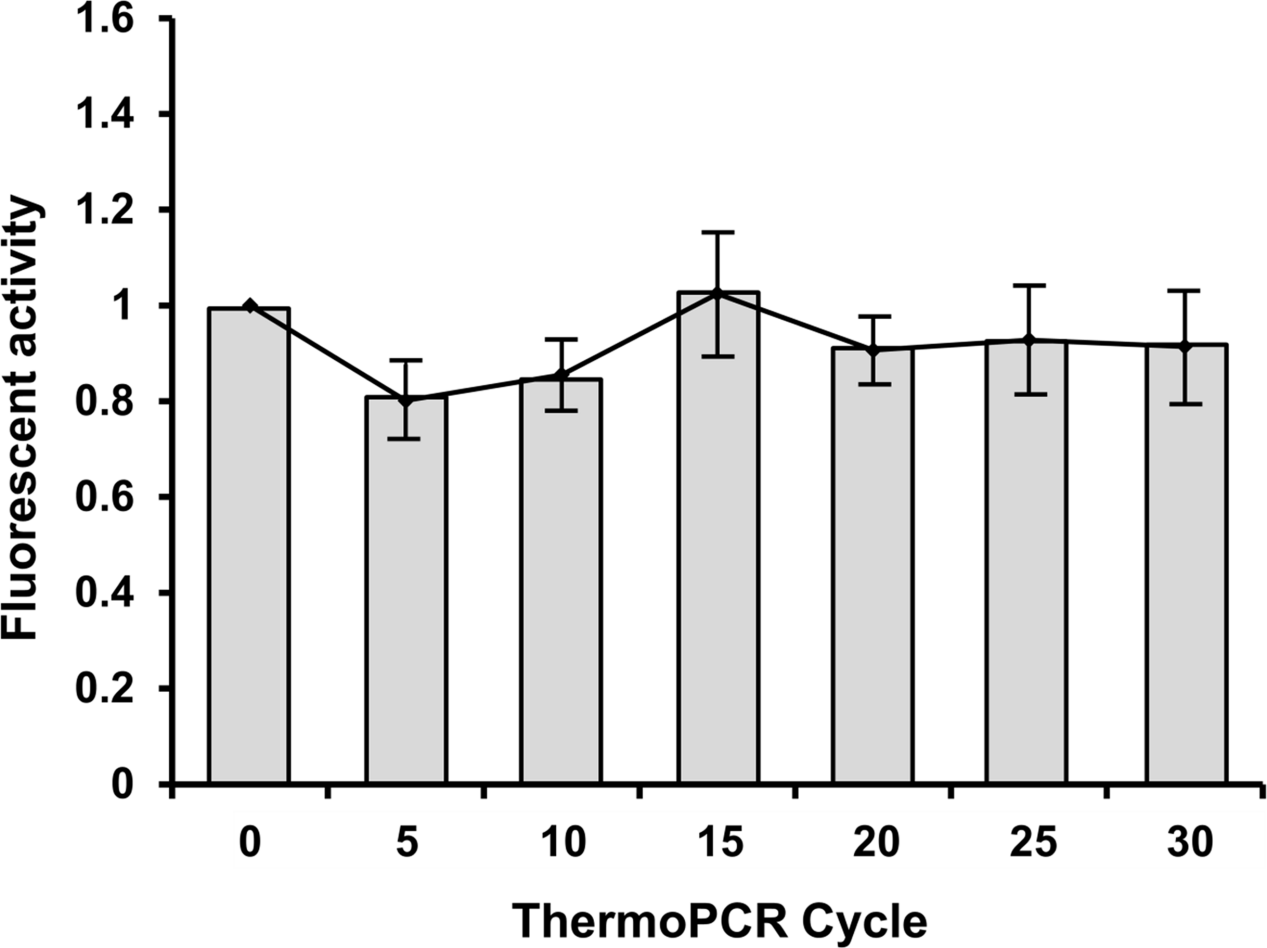
Thermostability of Ap-contained oligo-microsphere.

The thermal stable attachment of DNA probes on the microspheres was investigated (see **Fig 4**). We produced two types of microspheres containing 5’acrydite-DNA probe (Ap) and unmodified DNA (un-AP) with identical sequences. Each group was subjected to a standard thermocycling program (5, 10, 15, 20, 25 and 30 rounds of amplification performed at 95°C for 30 s, 52°C for 30 s, and 72°C for 30 s) for PCR. Following each round of thermocycling, the microspheres were gently washed and incubated with a complementary Cy-3 labeled primer (cAp) to assess DNA loss during thermocycling. A constant result of DNA hybridization demonstrates a strong attachment of DNA in the Ap-microspheres. Background activity indicating non-specific interactions between unmodified DNA and polyacrylamide structures is not observed.

**Fig 5:**
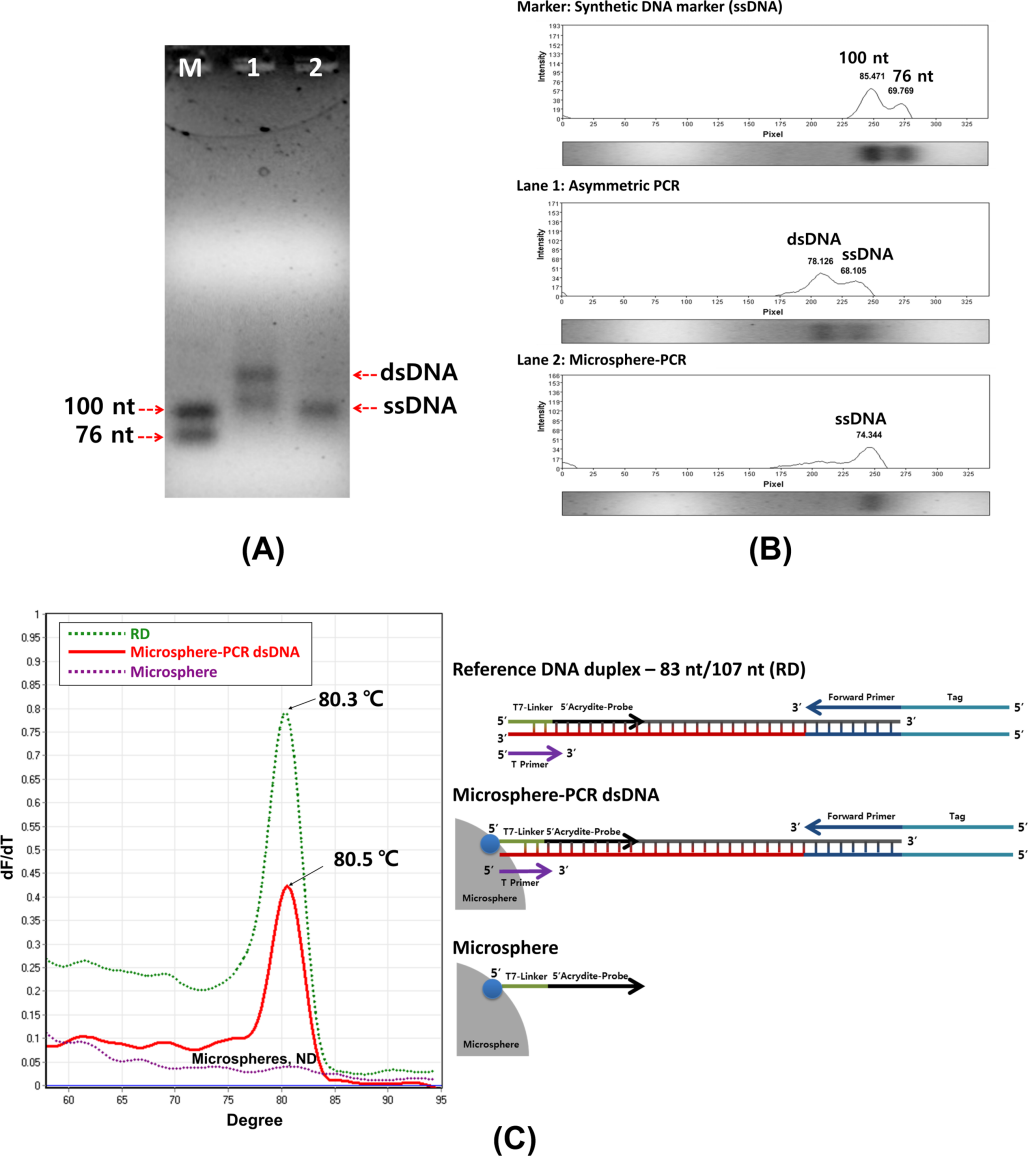
Microsphere-PCR for amplifying ssDNA. (A) The DNA amplicons were run on a 2% agarose gel followed by EtBr staining. (B) Agarose gel configuration to determine the relative location of PCR bands and their intensity from asymmetric PCR and microsphere-PCR experiments. M: Synthesized ssDNA Marker (76 nt, 100 nt), Lane 1: Asymmetric PCR (primer (F/R) ratio_20: 1), Lane 2: Microshperes-PCR. (C) Melting Tm analysis for synthetic DNA hybrids (Reference Duplex, RD) and Microsphere-PCR dsDNA byproducts. Co-polymerized Ap-microspheres was tested as a control. (ND: Not detected).

Co-polymerized Ap-microspheres were then applied to ssDNA amplification. **Fig 1** is shown to simply illustrate the use of co-polymerized Ap-microsphere in ssDNA amplification. Ap consisting of 18 nt-probe (AGATTGCACTTACTATCT), 7 nt-linker (TTTTTTT), and 5’-acrydite, provides a free-3'OH end for DNA polymerase and the annealing to their complementary sequence in template DNA (plus (+) strand, 76 nt). A detail sequences used to amplify ssDNA were listed in **Table 1**. Complementary DNA template (minus (-) strand) was precisely extended by adding a site-specific nucleotide. Their successful extension was confirmed by adding Cy-3 labeled forward DNA primer (data not shown). The DNA extended was itself used as a template for ssDNA aptamer amplification. To distinguish ssDNA amplicons (100 nt) from the initial template DNA (76 nt), the tag (24 nt)-forward primer was participated in microsphere-PCR. Complementary DNA extension and ssDNA amplification were performed in a single-tube reaction using Ap-microspheres, template, and forward primer, resulting in a streamlined microsphere-PCR protocol. Amplified products were directly loaded onto an agarose gel without additional strand-separation step such as dsDNA degradation or affinity purification. The amplified ssDNA was clearly demonstrated by comparing to known size markers (76 nt ssDNA and 100 nt ssDNA, synthetic DNA from Bioneer Co., Korea) run on 2% agarose gel electrophoresis analysis. In addition, dsDNA contaminant was not observed as shown in **Fig 5A and 5B**. Lane 1 shows asymmetric PCR products containing dsDNA and ssDNA. Lane 2 products were ssDNA by microsphere-PCR. Gel Doc™ EZ data provided agarose gel configuration to determine the relative location of PCR bands and their intensity from asymmetric PCR and microsphere-PCR experiments. To verify the formation of dsDNA duplex, the real-time PCR amplification and melting Tm analysis were performed by using specific primer sets (T primer and Tag Forward primer, TFp). The results in **Fig 5 C** show that the microsphere-PCR dsDNA presented a temperature for the melting of 80.5°C. There were no significant differences between the Tm observed for reference duplexes (RD, 80.3°C).

Asymmetric PCR is one of the methods used to generate ssDNA. This method uses non-equal amounts of reverse and forward primer (e.g. 20: 1 primer ratio) to generate large amounts of ssDNA together with a small fixed amount of dsDNA. Although the conventional asymmetric PCR generates a single-stranded template, it is inefficient and difficult to optimize each reaction to yield adequate and consistent ssDNA. In addition, dsDNA byproducts must be removed. **Fig 5** shows a comparison result between the conventional asymmetric PCR and our microsphere-PCR. The microsphere-PCR makes efficient use of a single primer to produce ssDNA based on co-polymerized Ap-microspheres, thus promoting accumulation of ssDNA in aqueous phase. Since dsDNA byproducts were predominantly attached to the surface of functional microsphere supporters during amplification, ssDNAs could be collected by simple pipetting from the PCR reaction tube.

To date, several strategies have been developed to amplify ssDNA that could help reduce DNA SELEX rounds by improving the quality of random sub-pools of single stranded oligonucleotides (ssDNA). SsDNA amplification by asymmetric PCR is one critical step in the traditional DNA SELEX procedure. It can affect the selection of highly specific candidates from the DNA random library. In addition, although the development of technical methods for generating ssDNA has helped us to increase the efficiency of sample throughput, processing bottleneck still remains at the separation step due to high costs and incomplete purification yields from dsDNA by-products. To generate ssDNA without separation, we produced polyacrylamide microspheres to expose DNA probes based on co-polymerization method. This attachment technology was easily adapted by using the droplet-based microfluidic platform, in terms of on-flow polymerization of oligo-microspheres. Polymer microspheres having nearly uniform diameters were successfully produced using this microfluidic platform. The exposure of oligonucleotides was assessed using fluorescence-attached oligonucleotides. Based on co-polymerized 5’-Ap-microspheres, microsphere-PCR was attempted in this work. Complementary DNA extension and ssDNA amplification were successfully performed in a single-tube reaction using Ap-microspheres, template, and forward primer, promoting the accumulation of ssDNA copy in the aqueous phase.

## Conclusion

We demonstrated that polyacrylamide microspheres bearing ssDNA probes could be readily synthesized through a microfluidic method. We also have shown how functionality, such as the co-polymerizable property, can be readily imparted to the ssDNA synthesis. This study provides a streamlined microsphere-PCR protocol in a single-tube reaction using only forward primer. To the best of our knowledge, this is the first report to demonstrate the application of microfluidic polymerization for ssDNA amplification. The microsphere-PCR method described herein can substantially improve the synthesis of ssDNA. The co-polymerizable oligo-microspheres is constructed here, making it available for use in assay development, DNA sequencing analysis and automatic DNA SELEX unit as well as customized ssDNA amplification kit.
